# Radiomics-Based Features for Prediction of Histological Subtypes in Central Lung Cancer

**DOI:** 10.3389/fonc.2021.658887

**Published:** 2021-04-29

**Authors:** Huanhuan Li, Long Gao, He Ma, Dooman Arefan, Jiachuan He, Jiaqi Wang, Hu Liu

**Affiliations:** ^1^ Department of Radiology, The First Hospital of China Medical University, Shenyang, China; ^2^ College of Computer, National University of Defense Technology, Changsha, China; ^3^ Sino-Dutch Biomedical and Information Engineering School, Northeastern University, Shenyang, China; ^4^ Imaging Research Division, Department of Radiology, University of Pittsburgh, Pittsburgh, PA, United States

**Keywords:** central lung cancer, histological subtype, computed tomography, radiomics, neural network

## Abstract

**Objectives:**

To evaluate the effectiveness of radiomic features on classifying histological subtypes of central lung cancer in contrast-enhanced CT (CECT) images.

**Materials and Methods:**

A total of 200 patients with radiologically defined central lung cancer were recruited. All patients underwent dual-phase chest CECT, and the histological subtypes (adenocarcinoma (ADC), squamous cell carcinoma (SCC), small cell lung cancer (SCLC)) were confirmed by histopathological samples. 107 features were used in five machine learning classifiers to perform the predictive analysis among three subtypes. Models were trained and validated in two conditions: using radiomic features alone, and combining clinical features with radiomic features. The performance of the classification models was evaluated by the area under the receiver operating characteristic curve (AUC).

**Results:**

The highest AUCs in classifying ADC vs. SCC, ADC vs. SCLC, and SCC vs. SCLC were 0.879, 0.836, 0.783, respectively by using only radiomic features in a feedforward neural network.

**Conclusion:**

Our study indicates that radiomic features based on the CECT images might be a promising tool for noninvasive prediction of histological subtypes in central lung cancer and the neural network classifier might be well-suited to this task.

## Introduction

Lung cancer (LC) is the leading cause of cancer-related deaths worldwide (1). Central LC is defined as tumors originating from the bronchial lumen or wall that usually occur in the segmental or more proximal bronchi (2). The central LC originates from primary, secondary, and tertiary bronchus, distinguishing it from peripheral LC that originates from bronchioles or further distance. Thus, central LC is close to the lung hilum, associated with higher mortality and morbidity due to it’s more likely to invade the mediastinum and main blood vessels. The LC can be divided into two main histological categories: small cell lung cancer (SCLC, ~25%) and non-small cell lung cancer (NSCLC, ~75%), the NSCLC can be further divided into two most common histological subtypes: adenocarcinoma (ADC) and squamous cell carcinoma (SCC) (3). SCC and SCLC are more common subtypes of central LC than ADC (4, 5). Histological classification of LC provides important information about tissue characteristics, which could determine the optimal treatment and/or therapy strategies for LC patients (6, 7). However, most central LC cases are unresectable when diagnosis, and CT-guided needle biopsy or bronchoscopy are frequently either unfeasible or unsuitable due to lesions adjacent to main blood vessels or bronchial obstruction (8). Moreover, central tumors are often heterogeneous manifestations, and histopathological samples may therefore be less reliable. Based on these limitations, non-invasive and accurate histological classification for LC patients demands special care.

In clinical practice, contrast-enhanced CT (CECT) is the main imaging modality used to evaluate LC. CECT images have been widely used to estimate the relationship between imaging characteristics and histopathological information in tumors (9). Radiomics, a high throughput data mining approach, can exploit the non-invasive medical image data (10). It focuses on extracting a large number of quantitative imaging features, which can provide a detailed and comprehensive characterization of the tumor subtypes (11). Recently, radiomic signatures from CT images have been used as a significant classification biomarker for LC (12–14). The radiomics might help to uncover tumor characteristics that are not easily appreciable by the naked eye. Some other researches focused PET-based radiomics to predict the histological subtypes of lung cancer (15–17). These researches selected patients with non-small cell lung cancer or all lung cancer subtypes as target population. Few studies focused on central LC’s association with radiomic features and histological subtypes. In this paper, we analyzed and classified central LC subtypes *via* machine learning classifiers diagnosed from CECT images.

## Materials and Methods

### Patients

This retrospective analysis study was ethically endorsed and approved by our institutional reviewing board with a waiver of the need for informed consent. Since standard definitions of central and peripheral locations of lung tumors varied within past literatures, we selected on representative method proposed by a previous study (18) to define the central tumors for our study. Central tumors were defined as the center of mass was within the hilar structures and peripheral tumors as the center of mass was within the parenchyma, with zero minimal contact with hilar structures. Patients with central LC who received blind diagnosed by two experienced radiologists (H. Li and H. Liu with 10 years of experience in lung CT interpretation) between January 2014 and June 2019 were selected for the cohort. Their histopathological subtypes were confirmed by surgical resections or bronchoscopies with transbronchial biopsies in our hospital. 513 such patients were selected from our institution’s database. Demographics (age, gender), smoking histories, and aggressive cancer characteristics (pleural effusion and pericardial effusion viewed from CT images) were selected as clinical features for further diagnostic analysis. Exclusion criteria chosen by our radiologists included: (1) patients received LC treatments; (2) unsatisfactory image quality due to severe artifacts; and (3) absent of contrast-enhanced CT imaging. A total of 200 patients with central LC were such screened for further processing (i.e. ADC: 55 patients; SCC: 66 patients; SCLC: 79 patients) (detailed clinical features see **Table 1**).

**Table 1:** Summary of the patient data in our cohort.

	Cohort			Total	*P* values
	ADC	SCC	SCLC (79)		
Age, mean±SD (years)	60.29±9.67	62.61±6.32	60.82±9.98	61.26±8.85	0.603
Gender, no. (%)					0.001
Male	33 (16.50%)	59 (29.50%)	53 (26.50%)	145 (72.50%)	
Female	22 (11.00%)	7 (3.50%)	26 (13.00%)	55 (27.50%)	
History of smoking, no. (%)					<0.001
yes	14 (7.00%)	61 (30.50%)	33 (16.50%)	108 (54.00%)	
no	41 (20.50%)	5 (2.50%)	46 (23.00%)	92 (46.00%)	
Pleural effusion, no. (%)					0.274
yes	14 (7.00%)	14 (7.00%)	26 (13.00%)	54 (27.00%)	
no	41 (20.50%)	52 (26.00%)	53 (26.50%)	146 (73.00%)	
Pericardial effusion, no. (%)					<0.001
yes	5 (2.50%)	2 (2.50%)	27 (13.50%)	34 (17.00%)	
no	50 (25.00%)	64 (32.00%)	52 (26.00%)	166 (83.00%)	

SD, standard deviation; ADC, adenocarcinoma; SCC, squamous cell carcinoma; SCLC, small cell lung cancer.

### CT Scanning Parameters

Patients included in this study were scanned as part of a routine clinical examination. Dual-phase chest CECT scans were acquired using various CT machine manufacturers including GE, Phillips, Siemens, and Toshiba. The acquisition parameters were set consistently as follows: Voltage 120 kVp (range 100–140 kVp), exposure time 751 ms (range 500–1782 ms), tube current 333 mA (range 100–752 mA, the thickness of the slice 1.0 mm (range 1.0 mm-2.0 mm). Although various contrast agents including Omnipaque, Isovue, Optiray, and Ultravist were used for the enhanced CT scans, contrast agent protocols (100 mL, iodide bromide 370 mg/mL at a rate of 2.5 mL/s) were relatively constant throughout all scans. All scan-based protocols were triggered at 100 HU in the thoracic aorta with subsequent scanning of the enhancement phase approximately 30-35s delay after the trigger.

### Tumor Segmentation

All thinnest CT original data were loaded to the Picture Archiving and Communication System (PACS) and then transferred to PHILIPS post-processing workstation for further analysis by J. Wang (a radiographer). All arterial CECT scans were reconstructed into an image series by using a thin slice thickness (1 mm) and mediastinal convolution kernels (standard B). The ITK-SNAP software (www.itksnap.org) was used to perform manual region of interest (ROI) mapping in the arterial enhancement phase images in chest CECT due to its best discriminating ability in tumors and other tissues. When data was transferred to ITK-SNAP software, all the images were anonymity for all readers. A three-dimensional ROI mapping including whole tumor was performed by an experienced radiologist (H. Li with 10 years of experience in lung CT interpretation) and double-check by H. Liu (10 years of experience in lung CT interpretation). A lung window level (window width at 1500 HU and window level at -500HU) was set for the tumor segmentation. All ROIs carefully excluded nearby compressed lung tissues and mediastinal tissues of the tumors as much as possible on arterial phase CECT. Because all the tumorous lesions were near the hilum, some vessels could not be excluded completely in the contours.

### Feature Extraction

3D radiomic features were extracted from the segmented ROI images from the original images and corresponding ROI masks using the Pyradiomics package (Pyradiomics plugin to 3D Slicer, version 1.30, default parameters) (19). These features included first-order histogram features (n = 18), second-order texture features (i.e. gray level co-occurrence matrix [GLCM, n = 24], gray level dense matrix [GLDM, n = 14], gray level size matrix [GLSZM, n = 16], nearest neighbor gray tone difference matrix [NGTDM, n=5], gray level run length matrix [GLRLM, n=16]), and shape-based features (n = 14). In total 107 radiomic features were captured from whole-tumor volume for further processing. Median values were calculated to remove outliers and standardize data.

### Feature Selection and Modeling

**Figure 1:**
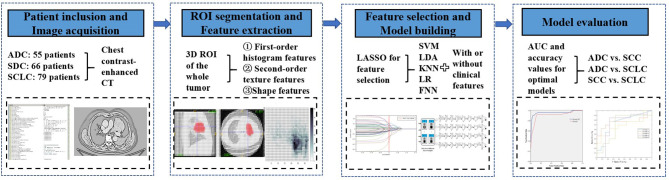
Workflow of classification analysis.

Five different machine learning techniques were used to classify histological subtypes of central lung cancer using radiomic features. They included Support Vector Machine (SVM) (the RBF kernel was used), Logistic Regression (LR), K-Nearest Neighbor (KNN), Latent Dirichlet Allocation (LDA), and feedforward neural network (FNN) (default Levenberg-Marquardt algorithm was used). FNN in this study has three layers: one input (whose neurons correspond to features), one hidden (with 10 hidden nodes), and one output (whose neurons correspond to subtypes). We applied the least absolute shrinkage and selection operator (LASSO) technique to select radiomic features with the strongest classification powers in the training set. After feature selection, a small set of selected features were used as the input of each classifier to classify ADC vs SCC, ADC vs SCLC, and SCC vs SCLC. The machine learning procedures were performed using MATLAB (Matlab R2016a; Mathworks, Natrick, Mass). We trained and validated the models in two conditions: using radiomic features alone (radiomic models), and combining clinical features (variables from **Table 1**) with radiomic features (integrated models) using 10-fold cross-validation. In this method, each time we select one fold as the testing set, the rest folds as the training set, thus the training set occupies 90% of the whole dataset each time. The workflow of classification analysis was shown in **Figure 1**.

### Statistical Analysis

Numerical data were expressed as means ± SDs. Inter-group differences in the mean values were assessed with t-test or chi-square test for clinical characteristics. The statistical significance was set at *p* < 0.05 (two-tailed). The interclass correlation coefficient was used to compare the consistency between the two radiologists. 30 randomly selected samples were outlined by our radiologists, and three radiomic features from three categories were selected to calculate the interclass correlation coefficient.

The diagnostic performance of models was evaluated by the area under the receiver operating characteristic (ROC) curve (AUC) and accuracy. 95% confidence intervals (CI) were calculated for the best AUC values. To obtain more stable performance, we used 10-fold cross-validation and reported the average performance of all folds. To obtain optimal models for each classification task, additional 100-round 10-fold cross-validations were followed for verification of the reproducibility of the predicted results.

## Results

### Patient Characteristics

The statistics of clinical features were shown in **Table 1**. These clinical features were included in the integrated model.

### Interobserver Agreement of Radiomic Features

The interclass correlation coefficients between the two radiologists ranged from 0.91 to 0.98, showing high interobserver agreement.

### The Optimal Models in Three Classification Tasks

**Figure 2:**
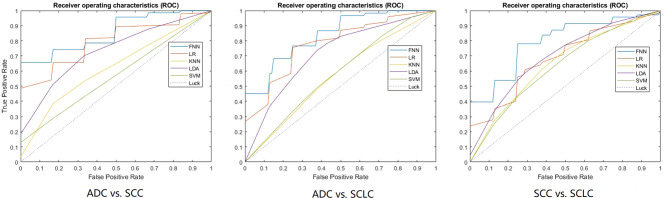
The ROC curves of the optimal model for each classification task. 10-fold cross-validation and five machine learning classifiers were utilized for predictive model construction in three tasks. In each ROC, the blue curve is the ROC of the model using the FNN classifier, the brown curve is the ROC of the model using the LR classifier, the yellow curve is the ROC of the model using the KNN classifier, the purple curve is the ROC of the model using the LDA classifier, and the green curve is the ROC of the model using the SVM classifier.

**Figure 3:**
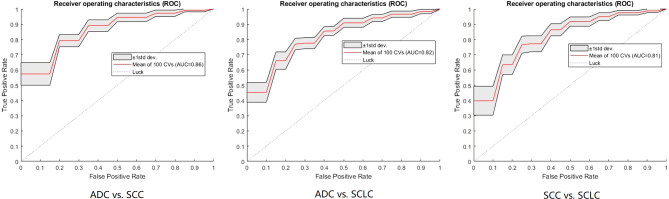
The ROC curves of the FNN model using 100-round cross-validations. In each task curve, the red curve is the mean ROC of the 100-round cross-validations and the shadow area is the standard deviation.

**Table 2** shows the models’ performance for classifying ADC vs SCC, ADC vs SCLC, and SCC vs SCLC, with and without clinical data, and using varying classifiers. The FNN classifier outperformed other classifiers in all three tasks using only radiomics features (The ROC curves are shown in **Figure 2**). The AUC and accuracy were 0.879 (95% CI: 0.826-0.931) and 70.6%, respectively, for classifying ADC vs. SCC. They were 0.836 (95% CI: 0.767-0.905) and 72.7%, respectively, for ADC vs. SCLC, and 0.783 (95% CI: 0.700-0.867) and 62.5%, respectively, for SCC vs. SCLC. Additional 100-round 10-fold cross-validations were performed for verification of the reproducibility of these results. The ROC curves of the 100-round cross-validations were reproducible in the FNN classifier of the three optimal models (**Figure 3**).

**Table 2:** The performance of different models in three classification tasks.

	ADC vs. SCC		ADC vs. SCLC		SCC vs. SCLC	
	AUC	accuracy	AUC	accuracy	AUC	accuracy
*Without clinical data*						
KNN	0.623	0.604	0.569	0.530	0.649	0.604
LDA	0.735	0.731	0.716	0.698	0.696	0.687
SVM	0.571	0.525	0.583	0.516	0.634	0.525
LR	0.795	0.575	0.778	0.545	0.686	0.587
FNN	0.879*	0.706	0.836*	0.727	0.783*	0.625
*With clinical data*						
KNN	0.524	0.487	0.565	0.570	0.534	0.570
LDA	0.716	0.708	0.735	0.723	0.641	0.630
SVM	0.571	0.525	0.574	0.504	0.503	0.457
LR	0.726	0.644	0.776	0.588	0.631	0.577
FNN	0.793	0.619	0.825	0.682	0.723	0.573

ROC, receiver operating characteristic; AUC, the area under ROC curve; ADC, adenocarcinoma; SCC, squamous cell carcinoma; SCLC, small cell lung cancer.

*means the highest AUC in each classification task.

For integrated models, our results showed that the AUC of clinical data was slightly lower than that of non-clinical data (for details, see **Table 2**).

## Discussion

Oncologists always seek to analyze the subtypes of cancer cells in LC patients. For unresectable tumors, the conventional method of biopsy (CT-guided needle or bronchoscopy) might not be an ideal option for the patient, since a pathological assessment performed on a specimen may be either unavailable or inaccurate. Thus, in this study, we tested an alternative computational method for histological subtyping in central LCs. Our results show that machine learning methods may have a potential capacity to subtype the central LC in cancer cells level prior to biopsies or operations. Among the five classifiers, FNN, a neural network-based approach, yielded the best results in the three classification tasks. Recently, deep learning methods have been widely applied in a variety of tasks and have outperformed many standard most classification and regression methods (20). Junior et al. utilized three machine-learning classifiers, showed that the highest testing performance in the histopathological pattern recognition of LC was obtained by neural networks (21). Our results combined with this previous study showed that the neural network classifier might display better performance than traditional machine learning in histological subtyping tasks.

In the classification of ADC vs. SCC and ADC vs. SCLC, the radiomic model showed a high performance (AUC of ADC vs. SCC: 0.879 and ADC vs. SCLC:0.836). The ADC subtype has distinct histologic characteristics with the other two subtypes. For example, ADC is associated with glandular architecture, whereas intercellular bridging and individual cell keratinization are prominent for SCC (4, 22). Moreover, the SCLC of neuroendocrine carcinoma whose cells have traits similar to those of nerve cells and hormone-producing cells (23), significantly differ from ADC. The underlying reason for the variability of radiomics features based on CECT may be correlated to biological heterogeneity within the tumor tissue. CT heterogeneity can be quantified using radiomics analysis, which reflects the coarseness and regularity that result from local spatial variations in image brightness. The spatial variations in image brightness may be enhanced by the intravenously injected contrast agent, which may result in the variable radiomics features (24, 25). Wu et al. extracted 440 radiological features from CT images of NSCLC to classify ADC vs. SCC. The accuracy was 0.7, and the AUC value was 0.72 (14). Haga et al. utilized a volume of interest method to analyze the subtype of early-stage non-SCLCs by different observers. The AUC values averaged over the different observers were 0.725 ± 0.070 (26). Zhu et al. used the LASSO logistic regression model to select five key features to construct the radiomic signature for histological subtype classification. The AUC of the radiomic signature to distinguish between lung ADC vs. SCC in validation cohorts was 0.893 (27). A multiphasic CECT study showed that the AUC of radiomics models in classifying ADC vs. SCLC were 0.857, 0.855, and 0.864 in non-enhanced, arterial phase, and venous phase, respectively (28). It is well known that histological tumor classification could arrange more detailed optimal treatment and/or therapy strategies for cancer patients in clinical practice. Recent advancement in LC therapies are characterized by the discovery of targetable mutations and histology-based therapeutic regimen selection (29, 30). For example, pemetrexed chemotherapy is the preferred treatment for stage IV lung ADC. More importantly, histology classification increases the likelihood of identifying patients with targetable mutations like EGFR mutations, which occur primarily in ADC (22). Our study combined with previous studies implies that noninvasive histology classification of ADC vs. the other two subtypes (i.e. SCC and SCLC) by radiomic features acquired from CECT images has promising clinical applications for oncologic diagnosis and treatment guidance.

Studies focused on classifying SCC vs. SCLC were rare. A previous study showed weak performance in classifying these two subtypes, in which the AUCs of the models in nonenhanced, arterial and venous phases were 0.657, 0.619, and 0.664 using supervised machine learning models (28). Our models also showed weaker performance in classifying SCC vs. SCLC than the other two classification models. The relatively lower AUCs of classifying SCC vs. SCLC can be attributed to the fact that SCC and SCLC share similar pathological structures; both have a dense tumor cell arrangement with few stromal components. Although the performance of classifying SCC vs. SCLC is not as strong as the other two classification tasks, our results utilizing neural network classifiers were still promising (AUC=0.783). As for the integrated models, we found that the AUCs with clinical data was slightly lower than that those non-clinical data, indicating that the addition of clinical and qualitative imaging factors to the predicting model did not significantly improve the model’s performance. It may be that these clinical characteristics are not significantly important for the predictive performance of the models. Some clinical features were selected by LASSO, but the corresponding performance decreased, suggesting that their combination weakens the model’s predictive performance due to data compatibility.

Our study has some limitations: First, we could not eliminate the possibility of including small regions of normal tissue or vessels in our segmentations. Additionally, our segmentations might be not completely reproducible due to variations in manual segmentation. In the future, an automated segmentation method should be applied to avoid this problem. Second, although we used a 10-fold cross-validation method, an independent cohort should be recruited for validation in the future. Third, the FNN method in this study was a primary neural network using three layers, and additional layers could yield improved results. Fourth, our relatively small sample size limited the expansion of the radiomic signatures, accordingly, a larger cohort should be recruited in future studies. Fifth, this study utilized a retrospective single-center research approach. Further investigation would benefit from forward-looking and multi-center approaches.

## Conclusion

In conclusion, the use of a radiomic approach for classifying the histological subtypes of central LC demonstrates the potential of radiomic features in differentiating central LC histology subtypes. The neural network classifier has a stronger effect on the aggregation of radiomic features based on the CECT than traditional machine-learning classifiers in histological subtyping tasks. Larger-scale studies are suggested to further validate the reproducibility and stability of these radiomics-based features extracted from CECT images.

## Data Availability Statement

The raw data supporting the conclusions of this article will be made available by the authors, without undue reservation.

## Ethics Statement

The studies involving human participants were reviewed and approved by The First Hospital of China Medical University. Written informed consent for participation was not required for this study in accordance with the national legislation and the institutional requirements.

## Author Contributions

Conception and design: HL and HM. Development of methodology: LG. Data Acquisition, analysis and interpretation: HHL, JH, and JW. Writing, review, and/or revision of the manuscript: HHL, DA, and HL. Study and supervision: HL. All authors contributed to the article and approved the submitted version.

## Funding

This work was supported by a grant from the National Natural Science Foundation of China (61702087).

## Conflict of Interest

The authors declare that the research was conducted in the absence of any commercial or financial relationships that could be construed as a potential conflict of interest.

## Abbreviations

LC, Lung cancer; SCLC, Small cell lung cancer; NSCLC, Non-small cell lung cancer; ADC, Adenocarcinoma; SCC, Squamous cell carcinoma; CECT, Contrast-enhanced CT; PACS, Picture archiving and communication system; ROI, Region of interest; GLCM, Gray level co-occurrence matrix; RLM, Run-length matrix; GLSZM, Gray level size matrix; LASSO, Least absolute shrinkage and selection operator; ROC, Receiver operating characteristic; AUC, Area under ROC curve; KNN, K-Nearest Neighbor; LDA, Latent Dirichlet Allocation; SVM, Support Vector Machine; LR, Logistic Regression; FNN, Feedforward Neural Network; SD, Standard deviation; CI, Confidence intervals.

## References

[1] Bhattacharjee A Richards WG Staunton J Li C Monti S Vasa P. Classification of Human Lung Carcinomas by mRNA Expression Profiling Reveals Distinct Adenocarcinoma Subclasses. Proc Natl Acad Sci USA (2001) 98(24):13790–5. 10.1073/pnas.191502998 PMC61120 11707567

[2] Moghissi K Dixon K Thorpe JAC Stringer M Oxtoby C. Photodynamic Therapy (PDT) in Early Central Lung Cancer: A Treatment Option for Patients Ineligible for Surgical Resection. Thorax (2007) 62(5):391–5. 10.1136/thx.2006.061143 PMC2117198 17090572

[3] Travis WD. Pathology of Lung Cancer. Clin Chest Med (2011) 32(4):669–92. 10.1016/j.ccm.2011.08.005 22054879

[4] Travis WD. Pathology of Lung Cancer. Clinics Chest Med (2002) 23(1):65–81, viii. 10.1016/S0272-5231(03)00061-3 11901921

[5] Schuurbiers OC Meijer TW Kaanders JH Looijen-Salamon MG de Geus-Oei L-F van der Drift MA. Span, Glucose Metabolism in NSCLC is Histology-Specific and Diverges the Prognostic Potential of 18FDG-PET for Adenocarcinoma and Squamous Cell Carcinoma. J Thoracic Oncol (2014) 9(10):1485–93. 10.1097/JTO.0000000000000286 25170642

[6] Tian S Wang C An MW. Test on Existence of Histology Subtype-Specific Prognostic Signatures Among Early Stage Lung Adenocarcinoma and Squamous Cell Carcinoma Patients Using a Cox-model Based Filter. Biol Direct (2015) 10:15. 10.1186/s13062-015-0051-z 25887039 PMC4415297

[7] Skrzypski M Dziadziuszko R Jassem E Szymanowska-Narloch A Gulida G Rzepko R. Main Histologic Types of non-Small-Cell Lung Cancer Differ in Expression of Prognosis-Related Genes. Clin Lung Cancer (2013) 14(6):666–73.e2. 10.1016/j.cllc.2013.04.010 23870818

[8] Travis WD Brambilla E Noguchi M Nicholson AG Geisinger K Yatabe Y. Diagnosis of Lung Adenocarcinoma in Resected Specimens: Implications of the 2011 International Association for the Study of Lung Cancer/American Thoracic Society/European Respiratory Society Classification. Arch Pathol Lab Med (2013) 137(5):685–705. 10.5858/arpa.2012-0264-RA 22913371

[9] Nguyen-Kim TD Frauenfelder T Strobel K Veit-Haibach P Huellner MW. Assessment of Bronchial and Pulmonary Blood Supply in non-Small Cell Lung Cancer Subtypes Using Computed Tomography Perfusion. Invest Radiol (2015) 50(3):179–86. 10.1097/RLI.0000000000000124 25500892

[10] Lambin P Rios-Velazquez E Leijenaar R Carvalho S van Stiphout RG Granton P. Radiomics: Extracting More Information From Medical Images Using Advanced Feature Analysis. Eur J Cancer (2012) 48(4):441–6. 10.1016/j.ejca.2011.11.036 PMC4533986 22257792

[11] Cufer T Ovcaricek T O’Brien ME. Systemic Therapy of Advanced non-Small Cell Lung Cancer: Major-Developments of the Last 5-Years. Eur J Cancer (2013) 49(6):1216–25. 10.1016/j.ejca.2012.11.021 23265700

[12] Ganeshan B Panayiotou E Burnand K Dizdarevic S Miles K. Tumour Heterogeneity in non-Small Cell Lung Carcinoma Assessed by CT Texture Analysis: A Potential Marker of Survival. Eur Radiol (2012) 22(4):796–802. 10.1007/s00330-011-2319-8 22086561

[13] Ganeshan B Abaleke S Young RC Chatwin CR Miles KA. Texture Analysis of non-Small Cell Lung Cancer on Unenhanced Computed Tomography: Initial Evidence for a Relationship With Tumour Glucose Metabolism and Stage. Cancer Imaging (2010) 10:137–43. 10.1102/1470-7330.2010.0021 PMC2904029 20605762

[14] Wu W Parmar C Grossmann P Quackenbush J Lambin P Bussink J. Exploratory Study to Identify Radiomics Classifiers for Lung Cancer Histology. Front Oncol (2016) 6:71. 10.3389/fonc.2016.00071 27064691 PMC4811956

[15] Hyun SH Ahn MS Koh YW Lee SJ. A Machine-Learning Approach Using Pet-Based Radiomics to Predict the Histological Subtypes of Lung Cancer. Clin Nucl Med (2019) 44(12):956–60. 10.1097/RLU.0000000000002810 31689276

[16] Han Y Ma Y Wu Z Zhang F Zheng D Liu X. Histologic Subtype Classification of non-Small Cell Lung Cancer Using PET/CT Images. Eur J Nucl Med Mol Imaging (2021) 48(2):350–60. 10.1007/s00259-020-04771-5 32776232

[17] Koyasu S Nishio M Isoda H Nakamoto Y Togashi K. Usefulness of Gradient Tree Boosting for Predicting Histological Subtype and EGFR Mutation Status of non-Small Cell Lung Cancer on (18)F FDG-PET/CT. Ann Nucl Med (2020) 34(1):49–57. 10.1007/s12149-019-01414-0 31659591

[18] Brooks DR Austin JH Heelan RT Ginsberg MS Shin V Olson SH. Influence of Type of Cigarette on Peripheral Versus Central Lung Cancer. Cancer Epidemiol Prev Biomarkers (2005) 14(3):576–81. 10.1158/1055-9965.EPI-04-0468 15767332

[19] van Griethuysen JJM Fedorov A Parmar C Hosny A Aucoin N Narayan V. Computational Radiomics System to Decode the Radiographic Phenotype. Cancer Res (2017) 77(21):e104–7. 10.1158/0008-5472.CAN-17-0339 PMC5672828 29092951

[20] LeCun Y Bengio Y Hinton G. Deep learning. Nature (2015) 521(7553):436–44. 10.1038/nature14539 26017442

[21] Junior JRF Koenigkam-Santos M Cipriano FEG Fabro AT de Azevedo-Marques PM. Radiomics-Based Features for Pattern Recognition of Lung Cancer Histopathology and Metastases. Comput Methods Programs Biomed (2018) 159:23–30. 10.1016/j.cmpb.2018.02.015 29650315

[22] Rekhtman N Ang DC Sima CS Travis WD Moreira AL. Immunohistochemical Algorithm for Differentiation of Lung Adenocarcinoma and Squamous Cell Carcinoma Based on Large Series of Whole-Tissue Sections With Validation in Small Specimens. Modern Pathol (2011) 24(10):1348–59. 10.1038/modpathol.2011.92 21623384

[23] Champaneria MC Modlin IM Kidd M Eick GN. Friedrich Feyrter: A Precise Intellect in a Diffuse System. Neuroendocrinology (2006) 83(5-6):394–404. 10.1159/000096050 17028417

[24] Perrin T Midya A Yamashita R Chakraborty J Saidon T Jarnagin WR. Short-Term Reproducibility of Radiomic Features in Liver Parenchyma and Liver Malignancies on Contrast-Enhanced CT Imaging. Abdominal Radiol (2018) 43(12):3271–8. 10.1007/s00261-018-1600-6 PMC6209534 29730738

[25] Bézy-Wendling J Kretowski M Rolland Y Le Bidon W. Toward a Better Understanding of Texture in Vascular CT Scan Simulated Images. IEEE Trans Biomed Eng (2001) 48(1):120–3. 10.1109/10.900272 11235585

[26] Haga A Takahashi W Aoki S Nawa K Yamashita H Abe O. Classification of Early Stage non-Small Cell Lung Cancers on Computed Tomographic Images Into Histological Types Using Radiomic Features: Interobserver Delineation Variability Analysis. Radiol Phys Technol (2018) 11(1):27–35. 10.1007/s12194-017-0433-2 29209915

[27] Zhu X Dong D Chen Z Fang M Zhang L Song J. Radiomic Signature as a Diagnostic Factor for Histologic Subtype Classification of non-Small Cell Lung Cancer. Eur Radiol (2018) 28(7):2772–8. 10.1007/s00330-017-5221-1 29450713

[28] Linning E Lu L Li L Yang H Schwartz LH Zhao B. Radiomics for Classifying Histological Subtypes of Lung Cancer Based on Multiphasic Contrast-Enhanced Computed Tomography. J Comput Assisted Tomography (2019) 43(2):300–6. 10.1097/RCT.0000000000000836 PMC6527094 30664116

[29] Manegold C. Treatment Algorithm in 2014 for Advanced non-Small Cell Lung Cancer: Therapy Selection by Tumour Histology and Molecular Biology. Adv Med Sci (2014) 59(2):308–13. 10.1016/j.advms.2014.08.008 25240504

[30] Barash O Peled N Tisch U Bunn PA Jr Hirsch FR Haick H. Classification of Lung Cancer Histology by Gold Nanoparticle Sensors. Nanomed: Nanotechnol Biol Med (2012) 8(5):580–9. 10.1016/j.nano.2011.10.001 PMC4745892 22033081

